# Using the *Drosophila* Transcriptional Reporter of Intracellular Calcium (TRIC) to examine lasting ethanol-induced changes in neuroexcitability

**DOI:** 10.17912/micropub.biology.000477

**Published:** 2021-09-23

**Authors:** Kahlan Merriman, Emily Petruccelli

**Affiliations:** 1 Southern Illinois University Edwardsville

## Abstract

The *Drosophila* transcriptional reporter of intracellular calcium (TRIC) is a genetic tool used to measure lasting changes in neuroexcitability. Both pan-neuronal and dopaminergic cells were examined with TRIC to test the hypothesis that ethanol exposure causes lasting changes in adult brain neuroexcitability. We found little to no impact on TRIC signal following acute and repeated ethanol vapor exposures. This work shows that TRIC may be useful in future investigations such as developmental or chronic drug exposure paradigms.

**Figure 1. Reporter of intracellular calcium following acute and repeated ethanol exposure:**
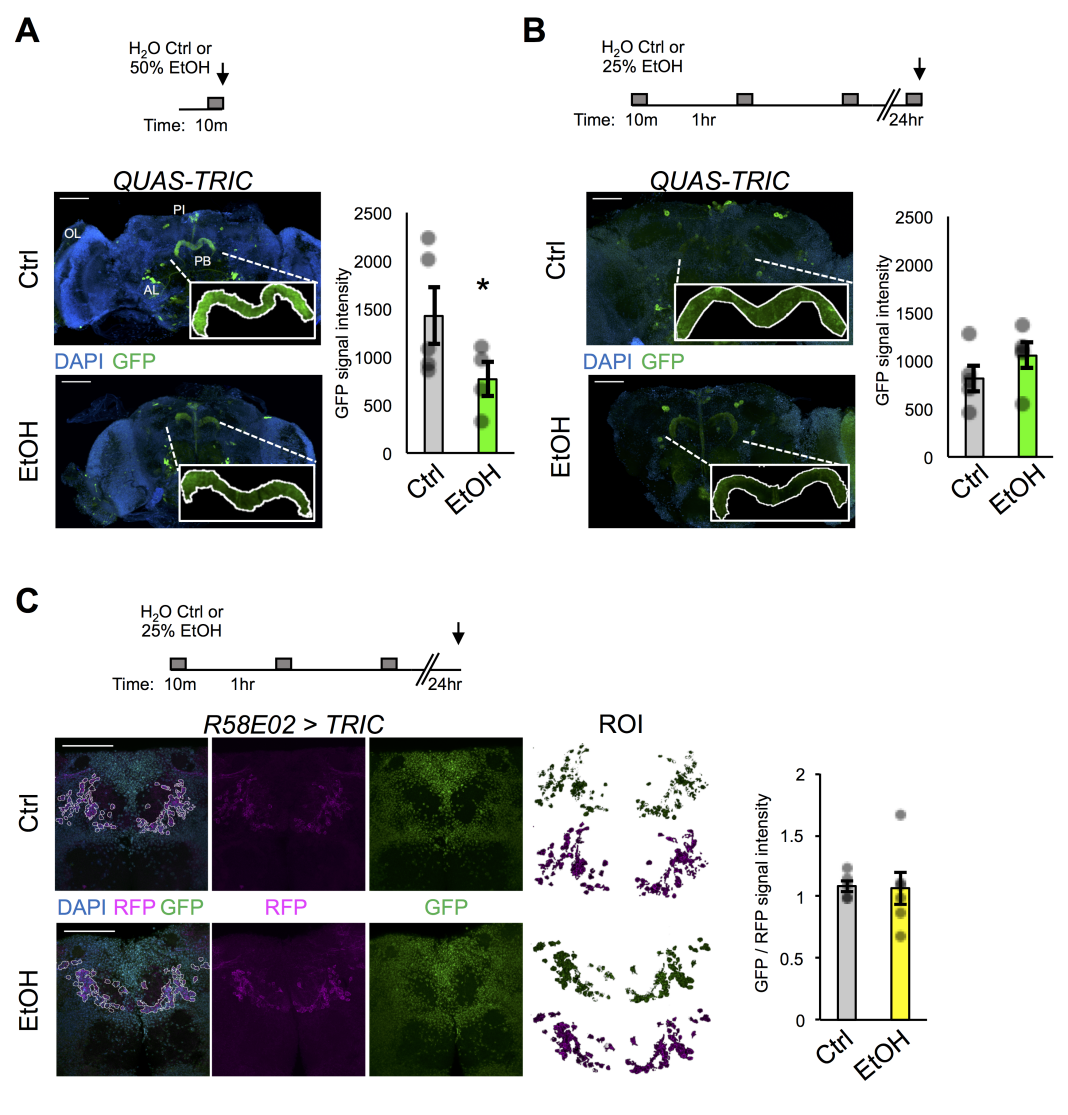
**A.***QUAS-TRIC* flies were fed QA and then received acute 10 min exposure to either H_2_O (Ctrl) or 50% EtOH vapor. Adult brain regions optic lobes (OL), antennal lobes (AL), pars intercerebrialis (PI), and protocerebral bridge (PB) are labeled in white. GFP signal, as measured by the mean pixel intensity within the PB, decreased upon ethanol exposure (Ctrl n = 5; EtOH n = 6). **B.** *QUAS-TRIC* flies were fed QA and then received repeated 10 min exposures to either H_2_O (Ctrl) or 50% EtOH vapor, followed by a 10 min exposure the following day. GFP signal in the PB was not significantly different in response to this repeated exposure paradigm (Ctrl n = 5; EtOH n = 5). **C.** Flies expressing TRIC in PAM dopaminergic neurons (*R58E02 > TRIC*) received repeated 10 min exposures to either H_2_O (Ctrl) or 50% EtOH vapor and were sacrificed the following day. Non-calcium-associated RFP signal was used to create a region of interest (ROI) and the ratio of mean GFP:RFP pixel intensity was quantified. No significant difference in PAM neuron TRIC signal was observed in response to this repeated exposure paradigm (Ctrl n = 5; EtOH n = 6). Black arrows in exposure paradigms represent the time of fly sacrifice. Bar graphs display the mean ± standard error of the mean of pixel intensity on a 16-bit scale (0-65536 a.u.); dots are raw data points. Student’s two-tailed t-test was used to determine statistical significance (*p < 0.05). Scale bars are 50 μm.

## Description

Alcohol Use Disorder (AUD) is associated with the disruption of various neurotransmitter systems including dopaminergic reward pathways (Banerjee 2014; Abrahao *et al.* 2017). For example, acute alcohol in low or moderate doses can potentiate or inhibit neuronal activity of distinct brain regions in the human central nervous system (Harrison *et al.* 2017). Similar observations have also been made in rodent AUD models, although different experimental approaches have highlighted the unresolved complexity of ethanol’s impact on neuroexcitability. For instance, low-ethanol-drinking mice showed increased ventral tegmental area (VTA) dopamine neuron firing, but surprisingly, high-ethanol-drinking animals displayed similar firing to that of naïve animals (Juarez *et al.* 2017). *Drosophila melanogaster* (flies) have also become an established model for the study of AUD, offering unbiased high-throughput screening, detailed behavioral analyses, and a platform for creating novel genetic tools (Devineni *et al.* 2011; Kaun *et al.* 2012). Intoxicated flies display acute ethanol-induced hyperactivity, loss of postural control, sedation, and develop both rapid and chronic tolerance. Furthermore, flies can make addiction-like reward memories of ethanol following repeated exposures (Kaun *et al.* 2011). To date, it remains unclear whether the long-term neuroexcitability of particular adult fly neurons are affected by acute or repeated ethanol exposure.

The transcriptional reporter of intracellular calcium (TRIC) is a recently developed genetic tool used to measure lasting changes in intracellular calcium, a common proxy that infers neuroexcitability (Grienberger and Konnerth 2012; Gao *et al.* 2015). TRIC genetics rely on the combination of tissue-specific binary expression systems (Gal4/UAS, QS/QF/QUAS, LexA/LexOp) and a split calmodulin (CamK) that ultimately induces a fluorescent protein to report intracellular calcium level (Gao *et al.* 2015). Thus, more intracellular calcium results in higher fluorescent signal and contrariwise. Unlike other well-known genetically-encoded calcium indicators, like GCaMPs, which detect rapid calcium flux at the millisecond level (Akerboom *et al.* 2013), TRIC signal reflects long-term fluctuations in neuroexcitability on the order of hours to days. For example, dark-rearing flies significantly reduced TRIC signal in the optic lobes and day-long-starved flies significantly reduced TRIC signal in insulin-producing pars intercerebrialis neurons (Gao *et al.* 2015). Pan-neuronal TRIC expression under the Q-system can be activated by feeding flies quinic acid (QA) to allow for temporal control of QUAS expression (Potter *et al.* 2010). Unlike the “QUAS-TRIC” flies, the TRIC system can also be tissue-specifically restricted, as well as contain a separate non-calcium-dependent fluorescent reporter for signal normalization in “UAS-TRIC” flies. For example, progeny from a cross of *NPF-Gal4* and *UAS-TRIC* lines show GFP signal corresponding to intracellular calcium levels and non-calcium-associated RFP background signal in neuropeptide F-producing neurons (Gao *et al.* 2015).

Here we use both QUAS-TRIC and tissue-specific UAS-TRIC lines to test the hypothesis that repeated ethanol exposures cause lasting changes to neuroexcitability in the adult fly brain. First, QUAS-TRIC flies were fed QA for three days to induce reporter system expression, then subjected to an either acute 10 min exposure to H_2_O (Ctrl) or 50% ethanol (EtOH) vapor, and finally sacrificed for brain dissection, immunohistochemistry, and confocal analysis. Similar to previously published work, strong GFP reporter signal under basal conditions was observed in various brain regions including the protocerebral bridge (PB), a region involved in locomotor control (Figure 1A) (Gao *et al.* 2015). Unexpectedly, though, little if any reporter signal was found in the optic lobes, despite flies being raised in standard 12 hr light / 12 hr dark conditions. However, TRIC signal was observed in the optic lobes upon pan-neuronal TRIC expression (*elav-Gal4 > UAS-TRIC*,not quantified here).

To determine if ethanol exposure altered basal QUAS-TRIC signal, we chose to quantify the mean pixel intensity of the PB. We reasoned that the PB might show ethanol-induced changes in neuroexcitability since flies visually showed locomotor impairment during the acute exposure. Interestingly, GFP reporter signal was significantly reduced following acute exposure to ethanol (Figure 1A), suggesting that acute ethanol decreases intracellular calcium in the PB. Because the TRIC signal would more likely be reliable in detecting lasting changes in intracellular calcium, we further hypothesized that repeated bouts of exposure would also reduce PB neuroexcitability. To test this, QUAS-TRIC flies were fed QA, then subjected to three repeated bouts of either H_2_O (Ctrl) or 50% ethanol (EtOH) vapor, and challenged again the next day with an acute exposure prior to analysis. This exposure paradigm was chosen because it most resembled the paradigm for when flies develop addiction-like reward memory of ethanol (Kaun *et al.* 2011). Surprisingly, no discernable difference in GFP reporter signal was detected between pre-exposed and mock-treated control flies (Figure 1B). This result appears to be due to a noticeable reduction in the control group’s signal intensity as compared to acute exposure. Together these findings suggest that prior intoxication experience had little to no impact on intracellular calcium within the PB or that the TRIC signal was too variable or inflexible to determine subtle ethanol-induced changes in neuroexcitability.

To further investigate whether pre-exposure to ethanol could influence neuroexcitability in particular neurons, we expressed the UAS-TRIC tool in reward-associated protocerebral anterior medial (PAM) dopaminergic neurons (*R58E02-Gal4*). The PAM neurons encode reward-associated memories and show heterogeneous basal activity that underlies bidirectional memory valence assignment (Liu *et al.* 2012; Yamagata *et al.* 2015) and are also required for making associative memories of ethanol (Scaplen *et al.* 2020). Therefore, we hypothesized that pre-exposure to ethanol would alter long-term PAM neuroexcitability. To test this, flies were either mock-treated or pre-exposed, then sacrificed the following day for analysis. For this experiment we chose to omit a challenge exposure because the onset or cessation of stimuli may act as punishment or reward. To quantify TRIC signal, the tissue-specifying RFP signal was used to create a region of interest and then calcium-reporting GFP intensity was normalized to the non-calcium-associated intensity of RFP. No detectable difference was observed in the overall GFP:RFP ratio in PAM neurons between mock-treated controls and pre-exposed flies (Figure 1C). These results suggest that prior intoxication experience had no overall effect on basal neuroexcitability of the reward-associated dopaminergic neuron cluster. The findings do not rule out the possibility that subsets of PAM neurons were differentially affected and future experiments could further investigate TRIC signal within other dopaminergic neurons, such as with TH-GAL4.

This work is, to the best of our knowledge, the first use of TRIC in the context of alcohol exposure. Although, we did not observe a dramatic impact on neuroexcitability in these experiments, there are many technical limitations to consider. For instance the intensity and stoichiometry of the TRIC components, the time and cell types of interest, and basal excitability state can all influence the range and accuracy of reporter detection. With these cautions in mind, TRIC may still offer a promising approach for future work investigating developmental exposure to ethanol or more chronic forced or volitional exposure paradigms.

## Methods

Fly husbandry and genetics

Fly stocks were maintained between 20-25°C, ~60% humidity, in 12hr light:12hr dark conditions, on Bloomington cornmeal-agar food. Male flies from 3-5 d old were used in every experiment. The pan-neuronal *elav^C155^-Gal4* (P{w[+mW.hs]=GawB} elav[C155] w[*]) and PAM dopaminergic *R58E02-Gal4* (*w[1118]; P{y[+t7.7] w[+mC]=GMR58E02-GAL4}attP2*) drivers were acquired from Dr. Karla Kaun’s lab (Brown University).

Full genotypes for TRIC lines acquired from Bloomington Drosophila Stock Center (Bloomington, IN, USA) are as follows:

**QUAS-TRIC (BDSC#61671):** w[*]; P{y[+t7.7] w[+mC]=nSyb-MKII::GAL4DBDo}attP24, P{w[+mC]=QUAS-p65AD::CaM}2, P{w[+mC]=UAS-mCD8::GFP.L}LL5/CyO; P{y[+t7.7] w[+mC]=nSyb-QF2.P}attP2, P{w[+mC]=tub-QS.P}9B

**UAS-TRIC (BDSC#61680):** w[*] P{y[+t7.7] w[+mC]=10XUAS-IVS-mCD8::RFP}attP18 P{y[+t7.7] w[+mC]=13XLexAop2-mCD8::GFP}su(Hw)attP8; P{y[+t7.7] w[+mC]=nSyb-MKIIK11A::nlsLexADBDo}attP24/CyO; M{w[+mC]=UAS-p65AD::CaM}ZH-86Fb

Quinic Acid (QA) Feeding

As previously demonstrated, QA relieves QS suppression in as little as 6 hr post feeding (Potter 2010). Here 75 mg of QA (Sigma-Aldrich, Product# 138622) was mixed with 300 µl of water and 2 drops of blue food-coloring were added to confirm ingestion. QA or control (H_2_O only) liquid was spread on top of food and allowed to absorb. Flies were placed on QA or control food for 3 d before being sacrificed for IHC.

For 10 min acute or challenge exposures, flies were transferred to empty vials, allowed to acclimate for ~5 min, then 500 µl of either 50% ethanol or control (H_2_O) was pipetted to the top of the cotton ball; vials were quickly sealed with parafilm for ~10 min prior to IHC. Pre-exposure was performed by transferring flies to perforated vials containing 1% agar and placed in one of two Rubbermaid containers outfitted with vapor input and output tubing; both containers received constant humidified air vapor, but one received 3 bouts of 10 min 50% ethanol vapor spaced by an hour each, while the other was a mock-treated control.

Immunohistochemistry (IHC)

IHC protocol was adapted from previous work (Petruccelli 2018). Briefly, flies were lightly anesthetized with CO_2_, brains dissected in PBS, fixed in 2% PFA PBST (Triton at 0.1% final concentration) for 1 hr at room temperature, and washed with PBST-Goat (NGS 0.5% final concentration) between incubations of the following primary (minimum 1:200 concentrations) and secondary (1:500) antibodies: Rabbit anti-GFP (Thermo Fisher Scientific, Cat# A11122); Chicken anti-RFP (Rockland Antibodies & Assays, Cat# 6000-901-379S); Goat AlexaFluor 488-conjugated anti-Rabbit (Thermo Fisher Scientific, Cat# A11008); Goat AlexaFluor 568-conjugated anti-Chicken (Thermo Fisher Scientific, Cat# A-11041). Brains were mounted in DAPI Fluoromount-G (SouthernBiotech, Cat# 0100-20).

Confocal Imaging and Analysis

Images were taken using an Olympus Fluoview (FV1000) confocal microscope and its accompanying software. Non-saturating gain and laser power were determined and held consistent within each experiment. At 20x or 40x oil objective, 2 µm and 1 µm slices were used, respectively. Imaging analysis was performed using FIJI (Schindelin *et al.* 2012). Max projection Z-stacks were created, regions of interest were determined, and pixel area and mean intensities were measured. Poorly dissected brains or brains with distinctly uneven staining were removed prior to quantitative analysis.

Statistics

Graphs were created in Microsoft Excel showing the mean, standard error of the mean, and raw data points. Pixel intensity values were statistically compared using a Student’s two-tailed T-test, *p<0.05.
